# Understanding and scientific progress: lessons from epistemology

**DOI:** 10.1007/s11229-022-03501-8

**Published:** 2022-02-17

**Authors:** Nicholas Emmerson

**Affiliations:** University of Birmingham, Department of Philosophy, ERI Building, Edgbaston, Birmingham, B25 2TT West Midlands UK

**Keywords:** Scientific progress, Understanding, Knowledge, Justification

## Abstract

Contemporary debate surrounding the nature of scientific progress has focused upon the precise role played by justification, with two realist accounts having dominated proceedings. Recently, however, a third realist account has been put forward, one which offers no role for justification at all. According to Finnur Dellsén’s (Stud Hist Philos Sci Part A 56:72–83, 2016) noetic account, science progresses when understanding increases, that is, when scientists grasp how to correctly explain or predict more aspects of the world that they could before. In this paper, we argue that the noetic account is severely undermotivated. Dellsén provides three examples intended to show that understanding can increase absent the justification required for true belief to constitute knowledge. However, we demonstrate that a lack of clarity in each case allows for two contrasting interpretations, neither of which serves its intended purpose. On the first, the agent involved lacks both knowledge and understanding; and, on the second, the agent involved successfully gains both knowledge and understanding. While neither interpretation supports Dellsén’s claim that understanding can be prised apart from knowledge, we argue that, in general, agents in such cases ought to be attributed neither knowledge nor understanding. Given that the separability of knowledge and understanding is a necessary component of the noetic account, we conclude that there is little support for the idea that science progresses through increasing understanding rather than the accumulation of knowledge.

## Introduction

In his 2006 paper, “Is Understanding a Species of Knowledge?”, Grimm notes a striking division between philosophers of science and epistemologists regarding their respective characterisations of *understanding*. On the one hand, there had been relative consensus among philosophers of science that understanding is merely a species of knowledge*,* knowledge which stands in some privileged relation to explanation.^1^ On the other, according to Grimm: ‘virtually every major epistemologist… has come to the conclusion that understanding is *not* a species of knowledge’ (2006: p. 516).^2^

Since 2006, however, the landscape of debate surrounding understanding has changed dramatically, with several leading philosophers of science having broken ranks.^3^ One area where this shift has had significant impact is in contemporary debate upon the nature and scope of *scientific progress* (SP)*.* In recent years, discussion surrounding SP has focused upon the precise role played by *justification*, with two contrary realist accounts dominating proceedings.^4^

According to the *semantic* account, most closely associated with Popper (1962, 1972) and Niiniluoto (1984, 1987, 1999, 2014), progress is made when scientific theories become closer to the truth, that is, when they become more verisimilar or approximately true.^5^ According to Bird’s (2007, 2008, 2019) *epistemic* account, on the other hand, science progresses through the accumulation of knowledge; true belief, justified by reliable scientific methodology.^6^ Where these perspectives diverge is in the extent to which justification is considered *constitutive* of progress.^7^ Both sides agree, however, that ‘beliefs without any justification simply do not belong to the scope of scientific progress’ (Niiniluoto, 2014: p. 76).

It ought to come as some surprise then, that in several recent papers, Dellsén (2016, 2017, 2018) has put forward a third realist account of SP, which does away with the notion of justification all together. According to this *noetic* account, science progresses when understanding increases, that is, when scientists grasp how to correctly explain or predict more aspects of the world that they did before (Dellsén, 2016).

In arguing that science progresses through understanding *rather than* knowledge, Dellsén is committed to a rejection of the orthodox view among philosophers of science, that ‘all (genuine as opposed to apparent) understanding is also knowledge’ (Bird, 2007: p. 84). Rather, Dellsén claims that understanding is distinct from knowledge in not requiring justification to be attained. It thus follows from Dellsén’s position that beliefs without any justification *do* belong in the scope of scientific progress.

The principal aim of this paper is to show that this controversial position of Dellsén’s is undermotivated, if not outright untenable. Dellsén (2016, 2017) provides three apparent examples of understanding without justification. The first two are hypothetical cases concerning Alice, a struggling student, and Bernie, a retired mechanic. The third example comes from the history of science itself: Einstein’s (1905/1956) explanation of Brownian motion.

What links these scenarios is that the agents involved supposedly come to understand a given phenomenon despite being in possession of evidence which ‘undermines the justification for their respective beliefs’ (Dellsén, 2017: p. 9).^8^ It is our belief, however, that these cases are in fact analogues of the Comanche-style cases first introduced by Kvanvig (2003). While this might not seem immediately problematic, we shall argue that concerns ought to arise once we consider that Comanche-style cases are rendered wholly unconvincing by analysis put forward by Grimm (2006).

In the next section we provide a more detailed account of Dellsén’s position, and how his three examples are supposed to support the thesis that understanding is separable from knowledge and that SP tracks the former rather than the latter. In Sect. 3 we examine Kvanvig’s (2003) Comanche case as well as Grimm’s (2006) criticism of it. Such cases suffer from a lack of clarity and, according to Grimm, once they are properly fleshed out two contrary interpretations of these scenarios emerge.

The problem for Dellsén is that neither interpretation proves that understanding and knowledge are separable: ‘on any way of filling out the details knowledge and understanding seem to sway together’ (Grimm, 2006: p. 520). In Sects. 4 and 5, we then show how this same argument can be levelled against Bernie’s case and Alice’s case, respectively. In the final two sections of this paper, we turn to Dellsén’s third example.

In Sect. 6, we argue that Einstein’s explanation of Brownian motion, like that of Alice and Bernie, is an analogue of Kvanvig’s Comanche case and thus fails to draw any distinction between understanding and knowledge. However, we also show that a comparison between Einstein’s case and recent research on SARS-COV-2, by Lourenço et al. (2020), provides novel insight into Comanche-style cases more generally. In Sect. 7 we highlight two fundamental problems with the noetic account of SP, raised by this comparison.

These problems suggest that it is Grimm’s first interpretation which ought to be adopted with respect to Comanche-style cases in general, and that neither understanding nor knowledge can be attributed to agents in such cases. Given that the separability of knowledge and understanding is a necessary component of Dellsén’s view, we conclude that there is little support for the idea that science progress through increasing understanding *rather than* the accumulation of knowledge.

## Understanding without knowing

It is interesting to note that, unlike knowledge, understanding is not an intrinsically realist notion. In recent work, for example, de Regt (2015, 2016) has argued for an *anti*realist notion of understanding which is not even moderately factive.^9^ In contrast, Dellsén is keen to maintain his realist convictions and thus takes understanding to be *quasi*-factive, suggesting that ‘the explanatorily/predictively essential elements of a theory must be true in order for the theory to provide grounds for understanding’ (2016: p. 73, fn6).^10^

Dellsén argues that his noetic account of SP, being based upon such a notion, can easily accommodate the realist mantra that progress is made by getting closer to the truth, since ‘one’s degree of understanding would simply be determined, at least in part, by how close to a fully correct representation of something is used for explanatory and predictive purposes’ (2018: p. 10).^11^ Consequently, Dellsén argues that it is *justification*, rather than truth, which stands to distinguish understanding from knowledge.^12^ In order to provide epistemological support for this idea, Dellsén (2017) introduces us to Alice, Bernie and Einstein.

Alice is a struggling student who has failed every assignment she has attempted this year. Despite this, Alice turns out to have an innate knack for geometry and successfully derives the Pythagorean theorem from a version of the original proof (without any help from a teacher or textbook). In this case, Dellsén argues, Alice does not have justification for believing that her proof is accurate, because her previous failures provide ‘good reasons to believe that *this* attempt at understanding a new subject matter in school is a failure as well’ (2017: p. 6). Having said this, it is clear, according to Dellsén, that Alice *understands* the Pythagorean theorem.

Bernie, on the other hand, is an automobile mechanic who reads in a newspaper that a convicted confidence trickster is coming to town. The newspaper article includes a picture of the man and a warning that not a single word he says can be trusted. Unfortunately for Bernie, the following day the man in question rings his doorbell and reports that his car has broken down. From the description of the car’s behaviour prior to the breakdown, provided by the trickster, Bernie *understands* that the issue is a broken timing belt. Nonetheless, Bernie is not justified in believing the issue to be a broken timing belt, since ‘he should know better than to trust a convicted con man’ (Dellsén, 2017: p. 8).

What is apparently peculiar about these cases, is that Alice and Bernie possess evidence which is not explanatorily relevant to the object of understanding which, nevertheless, undermines any justification they might have for their respective relevant beliefs. Dellsén does not limit his examples to hypotheticals, however. Indeed, being pulled from the annals of the history of science itself, his third example is perhaps his most convincing.

This case concerns Einstein’s (1905/1956) proposed explanation of Brownian motion in terms of the kinetic theory of heat, which was highly speculative at the time. As Einstein himself admits: ‘[i]t is possible that the movements to be discussed here are identical with the so-called “Brownian molecular motion”; however, the information available to me regarding the latter is so lacking in precision, that I can form no judgement in the matter’ (1905/1956: p. 1). As such, Dellsén (2016: p. 76) argues that Einstein, like Alice and Bernie, was clearly also lacking the justification required to *know* that the movements at issue were, in fact, real.

While Dellsén’s position is (to the best of our knowledge) unique among philosophers of science, the idea that understanding can be gained absent justification *does* find support within the epistemology literature. In the next section, we examine a case introduced by Kvanvig (2003) which bears a striking resemblance to the three cases appealed to by Dellsén.^13^ We also highlight convincing criticism of Kvanvig’s account provided by Grimm (2006). The problem for the noetic account of SP is that the examples used by Dellsén appear to be susceptible to this very same line of argument.

## Kvanvig’s comanche case

In *The Value of Knowledge and the Pursuit of Understanding* Kvanvig (2003) also argues for a realist notion of understanding. They suggests that, while the truth of one’s belief is an important factor for genuine understanding, the way in which one comes to those beliefs, their “*etiology*”, is irrelevant. To support this claim, Kvanvig provides an example which is analogous to the cases described by Dellsén (2017).

Suppose, Kvanvig (2003: pp. 197–198) argues, that we come to an understanding of *why* the Comanche dominated the Southern Plains of North America, through a textbook which we pick up from the local library. Now suppose that, while the textbook through which we gained this understanding is accurate, almost every other book on the topic is full of factual errors. Had we picked one of *these* books off the library shelf, instead of the accurate one, our beliefs about the Comanche would have been almost entirely false.

From this scenario, Kvanvig draws the same conclusion as Dellsén with respect to Alice and Bernie; while we do not have *knowledge* of the Comanche dominance of the Southern Plains, we do have the relevant *understanding*. As he explains:

It is the internal seeing or appreciating of explanatory relationships in a body of information which is crucial to understanding. When we think about knowledge, however, our focus turns elsewhere immediately, if we have learned our lessons from the Gettier literature: we think about the possibility of fortuitousness, or accidentality, of being right only by chance (Kvanvig, 2003: p. 198).

Yet, as Grimm (2006) highlights, there is a lack of clarity regarding exactly what is happening with respect to Kvanvig’s Comanche case. There appear to be two possible interpretations of the role of fortuitousness here. The first is that we form true beliefs based upon an unreliable *source* of information which just happens to get it right; ‘a crystal ball, a pathological liar, etc.’ (2006: p. 523). The second is that we form true beliefs on the basis of a reliable source of information in an *environment* in which the majority of possible sources are unreliable; ‘suppose by luck you happen across the only honest man in a room full of pathological liars’ (2006: p. 523).

If we are to follow Kvanvig, what links these interpretations is that wherever truth is accompanied by chance, understanding is possible, while knowledge is ruled out. According to Grimm, however, once these interpretations are fleshed out, it becomes apparent that knowledge and understanding are not so easily prised apart. On neither formulation, Grimm argues, does Kvanvig’s Comanche case provide an example of understanding absent knowledge.

In the first instance, taking the Comanche case to be the result of an unreliable source, Grimm (2006) argues that although knowledge doesn’t seem to be within reach, neither does understanding. Let us suppose that our textbook merely lays out the facts and leaves the insightful reader to construct explanations on their own: ‘the relevant accomplishment—the piecing together, as it were—would then be entirely internal [as Kvanvig claims]’ (Grimm, 2006: p. 526). Now imagine that the research undertaken by the author is ‘extremely shoddy… [conducted] on the basis of only one sample, and without controlling for the influence of other factors’ (Grimm, 2006: p. 526).

Grimm concludes that in such cases, even if it turns out that the information contained in the textbook *just happens* to be entirely correct, ‘it is hardly the case that the textbook reader who pieces together and develops an account of Comanche dominance would really come to understand *why* the Comanche dominated the Southern Plains’ (2006: p. 262).^14^ While we might concede that there is a *kind* of internal accomplishment in this case, it is not, according to Grimm, one of understanding: ‘[f]or that accomplishment, apparently, a stronger (alternatively: less accidental) connection to the Comanche is required’ (2006: p. 526).

In the second instance, taking Comanche cases to be the result of a reliable source located in an environment of unreliable sources, Grimm (2006) argues that while understanding seems to be within reach, so does knowledge. In this scenario we can assume that the author of our textbook is an expert on the Comanche, with impeccable research acumen, but that every other textbook on the topic which hits upon the truth does so only by accident. The key point to appreciate with this interpretation, according to Grimm, is that ‘Kvanvig overstates his case in claiming, without appropriate qualification, that knowledge is incompatible with luck’ (2006: p. 527).

A case highlighted by Hawthorne (2003) draws out the compatibility of knowledge with certain forms of luck here. Imagine that six children are each randomly assigned an atlas, all but one of which contains misinformation regarding the capital of Austria. Despite the fact that these books were assigned at random, Hawthorne (2003) argues, intuition would have it that the child whose book reads ‘Vienna’ *knows* which city is the capital of Austria. As Grimm explains:

[w]hen (as third party evaluators) we have reason to believe that the source of the information is good—here, that the textbook came from a reliable source etc—we tend to focus on the sense in which the belief is not lucky: it was no matter of luck, we think, that the textbook author identified Vienna as the capital of Austria, even if it was a matter of luck that this particular textbook ended up in the student’s hands’ (2006: p. 528).

Consequently, Grimm concludes that, in a scenario in which we are tempted to think that someone *understands* various things about the Comanche on the basis of a reliable source in an environment of unreliable sources, there is a strong tendency to suggest that the person also *knows* these things about the Comanche (2006: p. 529).

As we highlighted at the end of the previous section our central interest in Kvanvig’s Comanche case, and Grimm’s criticism of it, is the obvious structural similarity to the cases provided by Dellsén (2017). It is our contention that all three of Dellsén’s cases fall foul of the exact same problem facing the Comanche scenario, being similarly under-described.

Once fleshed out, it become apparent that none of these cases serve to sever the connection between understanding and knowledge, as Dellsén suggests. Just as we have seen with respect to the Comanche case, Alice’s, Bernie’s and Einstein’s cases can all be interpreted as instances where either knowledge *and* understanding are gained, or as instances where neither are. To see why, we shall begin by revisiting Bernie’s case in the next section, before returning to Alice’s case in Sect. 5, and Einstein’s case in Sects. 6 and 7.

## Bernie’s case revisited

As you will recall, Bernie (a retired mechanic) is in the unenviable position of being confronted with a known confidence trickster who describes the behaviour of his car, allowing Bernie to come to *understand* that the car in question has a broken timing belt. However, since ‘he should know better than to trust a convicted con man’, it appears that Bernie cannot *know* that the issue is a broken timing belt (Dellsén, 2017: p. 8).

At a first glance, Bernie’s case seems to be an obvious analogue of Grimm’s first interpretation of Kvanvig’s Comanche case. As Dellsén himself highlights, Bernie’s case ‘involves testimony from source that is known to be untrustworthy’ (2017: p. 8). Just as the author of the Comanche textbook *just happens* to provide accurate information despite unreliable methodology, Bernie’s con man *just happens* to provide accurate information regarding the behaviour of his car, despite the general tendency of con men to do quite the opposite.

In response to the Comanche case resulting from an unreliable source, Grimm concludes that neither understanding nor knowledge can be attributed to the reader of the textbook. While there may well be a *kind* of internal accomplishment is such cases, the accidental nature of the connection between the textbook and the Comanche suggests that this accomplishment is not understanding. Given the similarities between this case and Bernie’s, one might naturally suppose that the same conclusion can be drawn here.

In response to the fortuitous accuracy of the testimony provided by the con man, one might think that we can conclude that neither understanding nor knowledge should be attributed to Bernie either. In later sections, we shall argue that a thorough analysis of Einstein’s case suggests that this interpretation of Comanche-style cases is the correct one. However, for the time being, we wish to address an obvious response to this line of argument; that Bernie’s understanding in the case described by Dellsén appears to be far less accidental than the reader’s understanding in the analogous case described by Grimm.

While it is true that, in general, the testimony of a con man ought not be trusted, it is no mere accident that Bernie is able to understand the issue to be a broken timing belt. Bernie is, after all, a retired mechanic: an expert in his field. As such, one might think that Grimm’s argument doesn’t hold for Bernie. A closer inspection of the role of this expertise, however, reveals that this line of argument cannot save Dellsén’s claim that Bernie’s case serves to differentiate understanding from knowledge.

The importance of Bernie’s expertise to Dellsén’s argument can be seen if we consider an alternative scenario in which the con man describes the behaviour of his car to *us*, rather than to Bernie. Given that we are not even aware of where one might find a timing belt, let alone its function, it is patently obvious that any talk of understanding (or knowledge) is off the table in this case.

The understanding which Dellsén ascribes to Bernie, then, can only be the result of this expertise. As such, one might argue that Bernie *is* in possession of a certain type of understanding. It is presumably true of all retired mechanics (at least the competent ones), that they have an impeccable grasp of the *general* explanatory relations concerning the causes and symptoms of broken timing belts.^15^

The problem with this perspective is that, in shifting focus away from the fortuitous nature of the con man’s accurate testimony and towards the sense in which Bernie’s understanding is no mere luck, we run into Grimm’s second interpretation of Comanche-style cases. Here, of course, Grimm argues the expertise and reliable methodology of the author outweigh the luck in our having picked up the only accurate textbook.

As such, this interpretation pulls our intuition towards the ascription of both understanding *and* knowledge of the Comanche’s dominance of the Southern Plains. Similarly, any understanding which we attribute to Bernie based upon his expertise as a retired mechanic comes preloaded with *justification,* built up over a lifetime of first-hand experience dealing with similar issues in other vehicles.

On the face of it, it appears that Bernie’s case is a simple analogue of Grimm’s first interpretation of Kvanvig’s Comanche case, both of which result from an unreliable source. Given the fortuitous connection between the source of information and the broken timing-belt, one might conclude that neither knowledge nor understanding are present in this case.

However, there is a clear sense in which Bernie’s apparent understanding is no mere accident. One could argue that his expertise in automobile mechanics makes this example of Dellsén’s sufficiently robust to avoid this argument of Grimm’s. Although, just as the child’s knowledge survives the random assignment of an atlas which correctly labels the capital of Austria, in light of Bernie’s expertise, one might think that his own knowledge survives the fortuitous nature of the con man’s testimony.

Clearly, whether Bernie is taken to be capable of *both* understanding and knowledge or *neither*, will depend upon the extent to which Bernie’s expertise are thought to “trump” the fortuitousness of the con man’s testimony. What is important for our current purposes, of course, is that neither option suggests that Bernie’s case serves to sever the connection between understanding and knowledge: ‘on any way of filling out the details knowledge and understanding seem to sway together’ (Grimm, 2006: p. 520).

As we mentioned earlier, in Sects. 6 and 7, we shall argue that that there are pragmatic considerations which suggests that, in general, Comanche-style cases ought to be interpreted as instances of neither understanding nor knowledge. For the time being, however, we shall return to Alice’s case, and show that it too is analogous to Kvanvig’s Comanche case.

## Alice’s case revisited

To briefly refresh our memories, Alice is a student who manages to successfully derive the Pythagorean theorem using a version of the original proof. In light of Alice’s dismal track record of failure in previous school assignments, Dellsén argues that she is not justified in believing that her attempt at deriving the Pythagorean theorem is successful. As such, even though Alice ‘clearly’ understands the Pythagorean theorem she has no knowledge of it, lacking the requisite justification (Dellsén, 2017: p. 7).

It should come as no surprise that Alice’s case is open to the same interpretations as Bernie’s. The most obvious of these interpretations presents Alice’s case as an analogue of Grimm’s second interpretation of the Comanche case. Here, we might note that Alice comes to understand the Pythagorean theorem using ‘a version of Pythagoras’s original proof’ (Dellsén, 2017: p. 6). In this sense, it appears that the reliability of Alice’s source of information is beyond dispute, being Pythagoras himself.^16^

Just as when we focus upon the reliability of the author in the Comanche case, or on Bernie’s expertise, it could be argued here that Alice’s track record of academic failure is outweighed by the reliability of her information source. Thus, while we can grant that Alice understands the Pythagorean theorem, she also seems to *know* the Pythagorean theorem.

However, in response to a similar line of reasoning (put forward by an anonymous reviewer), Dellsén explicitly rejects this interpretation of Alice’s case. Instead, he provides an alternative description of Alice’s case which suggests that it ought to be interpreted in line with Grimm’s first interpretation of Kvanvig’s Comanche case:

we could easily stipulate that Alice, for whatever reason, got lucky in her attempt to prove the Pythagorean theorem and that she fails to construct similar proofs in geometry on other occasions (and/or that she would have failed to construct the proof of the Pythagorean theorem in most nearby possible worlds), in which case *her belief-forming process would not have been reliable* (Dellsén, 2017: p. 7, fn.10, italics are our own).

On Dellsén’s preferred interpretation then, we should take Alice herself to be the source of information in this case, with her track record of past failure in school assignments serving to establish Alice as an *unreliable source*. This reading, of course, makes Alice’s case analogous to the Comanche case where the author of the textbook bases their research upon extremely shoddy methodology.

In this instance, Grimm (2006) argues, even if the information contained in the textbook is correct, the reader of the textbook cannot be said to understand *why* the Comanche dominated the Southern Plains. It is clear to us that the same conclusion results from this interpretation of Alice’s case. To see why, we will need to briefly return to a specific aspect of Bernie’s case discussed above.

In the previous section, we saw that Bernie’s expertise (resulting from his being a retired mechanic) are of crucial importance to Dellsén’s claim that he can be said to understand the problem with the con man’s car. Imagining a scenario where *we* were in Bernie’s shoes, knowing practically nothing about the proper functioning of cars, any thought that we could be said to understand the problem is, quite clearly, off the table.

Suppose, however, that the con man pushes us to provide a diagnosis for his car’s issue (perhaps, somewhat ironically, he does not believe our protestations of vehicular ignorance and instead thinks us overly modest). Fortuitously, we had been watching a documentary about cars just that morning in which broken timing belts were mentioned, and since this is the first thing which comes to our minds, we put this forward as a possible explanation of the car’s behaviour prior to the breakdown.

The fact that our proposed explanation is correct here does nothing to alter our original verdict that we *do not* understand that the issue with the con man’s car is a broken timing belt. This situation clearly mirrors Alice’s. Just as we ‘got lucky’ in our attempt to explain the problem with the con man’s car, Alice ‘got lucky in her attempt to prove the Pythagorean theorem’ (Dellsén, 2017: p. 7, fn. 10). Given that understanding is quite clearly absent in the former case, there is little reason to think that Alice understands Pythagorean theorem in the latter.

The issue here, once again, is that it is simply not plausible to suppose that understanding is compatible with this level of epistemic luck. Recall that Dellsén suggests that Alice ‘would have failed to construct the proof of Pythagorean theorem in most nearby possible worlds’ (Dellsén, 2017: p. 7, fn. 10). This modal fragility might well seem wildly at odds with Dellsén’s further description of the *process* of Alice’s understanding, that she ‘grasped how to prove the Pythagorean theorem, realizing how later steps in the proof follow from earlier steps’ (Dellsén, 2017: p. 7, fn. 10).

If Alice truly grasps the interaction between these steps of her proof, it seems highly likely that she *would* be able to perform these same steps in nearby possible worlds. Putting aside the contradictory nature of these accounts of Alice’s case, this latter idea does not help Dellsén. Just as with Bernie’s case, in drawing our attention away from the sense in which Alice got lucky, we arrive back at the interpretation discussed at the beginning of this section (which Dellsén rejects), that Alice is in possession of both understanding *and* knowledge.

So far, we have argued that neither Alice’s case, nor Bernie’s, support the thesis that understanding is separable from knowledge. Given that the separability of these notions is a necessary condition of Dellsén’s claim that SP tracks understanding *rather than* knowledge, the noetic account certainly seems to be on shaky ground. Despite this, our task is not yet complete, since one example of Dellsén’s remains untested: Einstein’s use of the kinetic theory of heat to explain Brownian motion.

In the next section, we provide an analysis of this case, showing that it too is an analogue of Kvanvig’s Comanche case. What this means is that, like those cases described so far, Einstein’s case does not stand as a counterexample to the orthodox stance within philosophy of science, which holds that understanding is a species of knowledge. However, we also highlight an illuminating comparison between Einstein’s case and a recent SARS-COV-2 study by Lourenço et al. (2020). In Sect. 7, we show that this comparison suggests that Comanche cases ought to be interpreted in line with Grimm’s (2006) initial analysis, as instances of neither understanding nor knowledge.

## Einstein’s case revisited

As we saw in Sect. 2, Dellsén’s third case concerns Einstein’s (1905/1956) explanation of Brownian motion in terms of the kinetic theory of heat. Since the information available at the time was lacking, Dellsén (2016: p. 76) argues, Einstein was clearly also lacking the justification required to *know* that the movements at issue were, in fact, real.

Consequently, on the epistemic account, despite the fact that Einstein’s work on this topic ‘is widely considered to be one of the most significant achievements of one of history’s greatest scientists’ (Dellsén, 2016: p. 77), it does not count as progressive. The noetic account, on the other hand, can seemingly make sense of Einstein’s progress here, ‘because he enabled us to grasp how to correctly explain Brownian motion, thereby providing us with understanding of something that we were previously unable to understand’ (Dellsén, 2016: p. 76).

However, it is our contention that Einstein’s case, like Alice’s and Bernie’s, is open to two conflicting interpretations. One might think, given Einstein’s undeniable status as one of history’s greatest scientists, that he has the relevant expertise to warrant the ascription of understanding. Of course, in focusing upon the extent to which Einstein’s explanation was no mere fluke, we are driven towards the conclusion, as in Bernie’s case, that Einstein also *knew* Brownian motion to be explainable in terms of the kinetic theory of heat.

However, given the information available to Einstein in 1905, there is a very real sense in which he ‘got lucky’ in his explanation of Brownian motion. As Dellsén highlights, not only was Einstein’s information regarding Brownian motion lacking precision, but ‘[t]he kinetic theory of heat was very much up for debate at the turn of the twentieth century’ (2016: p. 76). In this sense, following Grimm’s (2006) reasoning, with respect to his first interpretation of the Comanche case, it might seem that we should attribute neither understanding nor knowledge to Einstein in 1905.

So far, we have not shown a preference for either of these interpretations. Such a stance is not, strictly speaking, necessary in order to show that Dellsén’s noetic account of SP is undermotivated. What matters for this argument is, as we have shown, that neither interpretation supports the thesis that understanding is separable from knowledge. However, we believe that a closer analysis of Einstein’s case suggests that it is Grimm’s first interpretation which should be adopted with respect to Comanche-style cases. Our reasoning here is based upon a particular feature of understanding recently highlighted by Wilkenfeld (2017).

In his 2017 paper ‘MUDdy understanding’, Wilkenfeld notes that ‘if we are not already in a situation where we know whether… the state of affairs represented by the explanans obtain, then we cannot know whether a particular explanation is *actually* conducive to maximal understanding’ (2017: p. 1290).^17^ In all three of the examples appealed to by Dellsén we know that the affairs represented by the explanans obtain.

In Alice and Bernie’s cases, this is simply a stipulation of the scenario. In Einstein’s case, we look back with the benefit of hindsight upon an explanation now universally accepted to be (at least approximately) true by the scientific community. However, when we consider an example where the accuracy of a given explanation is unknown, we come to realise that Comanche-style cases have only one viable interpretation.

Our chosen example concerns a recent study, carried out by Lourenço et al. (2020) at the University of Oxford, which caused something of a media frenzy, in reporting that half of the UK population could have been infected with SARS-CoV-2 as early as 19/03/2020.^18^ The authors of the study use the reported deaths from COVID-19, in the UK and Italy, to back-calculate the number of people infected with SARS-CoV-2.

As Hunter (2020) describes the study, it presents ‘an SIR (Susceptible-Infected-Recovered) model that [the authors] calibrate to the epidemic trajectory in both Italy and the UK using Bayesian approaches. Using a range of assumptions, they conclude that already a large proportion of the UK population, [possibly] up to 68% may already have been exposed to infection’.

However, this conclusion has met with some scepticism from the wider scientific community. According to Nairsmith (2020), for example, the study ‘rests on a key assumption which may or may not be the case’. Expanding upon this theme, Wood (2020) notes that ‘[t]he work merely makes assumptions about asymptomatic infection and mortality rates, but cannot measure them’. A further assumption made by the authors, highlighted by Gubbins et al. (2020), is the proportion of the population at risk of severe disease, a factor which is unknown.

Despite this, there does appear to be a relative consensus regarding the key take away from the study, which is, the need for more thorough serological studies in areas where epidemic spread has occurred. As Gubbins et al. (2020) puts it: the study’s conclusion is a *hypothesis*, not a fact: ‘a proper test will come from serological surveys [or large-scale surveys of virus genome diversity]—which will tell us how many people have been exposed.’

Like Einstein’s explanation of Brownian motion, the study conducted by Lourenço et al. (2020) puts forward an explanation which is compatible with, but underdetermined by, the evidence available at the time. Following Dellsén’s reasoning in Einstein’s case we can assume that, at time of writing, Lourenço et al. (2020) lack justification for believing that as much as 68% of the population of the UK had been infected with SARS-CoV-2 by 19/03/2020. What this means is that Lourenço et al. (2020), like Einstein in 1905, cannot *know* this to be the case and thus, on the epistemic account, this study does not count as progressive.

The key difference between these two cases is that the hypothesis put forward by Lourenço et al. (2020) remains just that: *a hypothesis*. At this point, we have no way of knowing how accurate their model is. Unlike the cases concerning Alice, Bernie and Einstein, we do not know that the states of affairs represented by the explanans obtain. As a result, it appears that we cannot make any pronouncement about the ascription of progress on the noetic account either. This is because Dellsén’s quasi-factive notion of understanding requires that ‘the explanatorily/predictively essential elements of a theory must be true’ (2016: p. 73 fn.6). It is precisely these explanatorily/predictively essential element which are called into question by Nairsmith (2020) and Wood (2003).

However, it is important to note that this is the same position in which we find Einstein in 1905. Just as we are currently unable to provide a verdict with respect to the progressive nature of the SARS-CoV-2 study, in 1905, it would have been impossible to provide a verdict with respect to Einstein’s explanation of Brownian motion. With the kinetic theory of heat being hotly contested at the time, and Einstein’s information regarding Brownian motion lacking precision, the verisimilitude of the explanatorily/predictively essential elements of this theory would be epistemically inaccessible.^19^

## The problem of epistemic access

The obvious next question concerns what it would take for us to be able to say whether or not the hypothesis put forward by Lourenço et al. (2020) constitutes SP. It appears that the answer to this question is the same for both the epistemic and noetic accounts. This situation would require what Gubbins et al. (2020) calls a ‘proper test’, that is, widespread serological, or virus genome diversity, surveys.

Let us call the time at which an original hypothesis is put forward *t*_h_ and the point at which a hypothesis is corroborated by evidence *t*_*c*_. Suppose that in several months, we arrive at *t*_*c*_, the results of the appropriate surveys are in, and we have confirmation of the accuracy of the assumptions made by Lourenço et al. (2020) at *t*_*h*_. At this point, Dellsén would presumably be happy to say that this study constitutes SP since, we would now be able to assert that Lourenço et al. (2020) provided a correct explanation/prediction despite, at the time, lacking justification for believing it.

The problem here, is that the results of future surveys which would vindicate their position, allowing Dellsén to grant that the explanatorily/predictively essential elements of their theory are true (and thus that theirs is a genuine case of SP), would also provide precisely the sort of *justification* which is required for SP on the epistemic account.

So, we are currently unable to ascribe either understanding or knowledge to Lourenço et al. (2020), and as a result, we cannot make a pronouncement as to the progressive (or otherwise) nature of their research according to either account of SP. However, the point at which we would be able to reasonably maintain that this piece of research constitutes scientific understanding, *t*_*c*_*,* is also the point at which we would be able to reasonably maintain that this piece of research constitutes scientific knowledge. Once again, knowledge and understanding sway together.

This same situation appears to hold for Einstein’s explanation of Brownian motion. Dellsén is in a position to grant that Einstein’s hypothesis constitutes progress only because it is now thought to be true. However, this situation is largely due to the later work of Perrin (1909), whose experimental verification of Einstein’s explanation of Brownian motion (among other advancements) earned him a Nobel Prize in 1926.

Perrin’s (1909) work plays the exact same role with respect to Einstein’s case as widespread serological or virus genome diversity surveys would with respect to the case of Lourenço et al. (2020)*:* providing *justification* for the belief that Brownian motion can be explained by the kinetic theory of heat. In 1905, when Einstein first put this hypothesis forward, we would have been unable to make a pronouncement as to the progressive nature of his proposed explanation. By the time the explanatorily/predictively essential elements of Einstein’s theory could reasonably be accepted as true, and thus qualify for understanding, they could also be justifiably believed; both elements being grounded in Perrin’s (1909) experimentation.

It would appear that, in order for us to be able to attribute understanding to the likes of Einstein (1905/1956) or Lourenço et al. (2020), we must first be in a position to assert that the explanatorily/predictively essential elements of the relevant theory are true. However, the truth of such elements will remain epistemically inaccessible until such time as confirmation, sufficient to supply epistemic justification, has been attained.

A natural response to this argument, would be to point out that even though it would have been impossible to make such a judgement at the time, the noetic account can still *retrospectively* assign understanding to Einstein, since we *now* know his explanation to be (a least approximately) true. The epistemic account can make no such claim, since in 1905 justification was lacking and, unlike understanding, justification cannot be retrofitted. However, it is difficult to see how this idea stays true to the central tenet of the noetic account of SP, that progress in science tracks understanding *rather than* knowledge.

If, as our argument suggests, Dellsén’s realist notion of understanding can only be ascribed *after* a theory has met with confirmation (and thus, justification) of some kind, the idea that understanding is playing the fundamental epistemic role here becomes a hard one to swallow. The case of Lourenço et al. (2020) highlights this nicely. When we think about what it would take for us to able to grant that this study constitutes progress, our intuitions pull us towards those criteria which would also provide justification for believing their hypothesis, i.e. widespread serological, or virus genome diversity, surveys. That this knowledge would allow us to retroactively assign understanding on behalf of Lourenço et al. (2020) at *t*_*h*_*,* does not change the fact that the tell-tale sign of progress in this case is *justification,* and thus*, knowledge*.

This issue also appears to raise another, concerning how the noetic account is to make sense of confirming instances themselves. According to Dellsén, remember, Einstein makes scientific progress in 1905 by proposing an explanation of Brownian motion in terms of the kinetic theory of heat. Although, confirmation of Einstein’s theory is not attained until around 1908. Clearly, this confirmation is of crucial importance to Einstein’s theory since, as we have already seen, it is Perrin’s (1909) work which allows for the ascription of knowledge at *t*_*c*_* and* Dellsén’s retroactive attribution of understanding at *t*_*h*_.

However, it is difficult to see how the noetic account can make sense of this, clearly progressive, step. After all, if science progresses through increasing understanding, and Einstein can be said to understand Brownian motion in 1905, what work is there left for Perrin’s experimentation to do? Verification constitutes a significant epistemic boon for Einstein’s theory; although, the noetic account of SP seems to be incapable of accounting for it: the proverbial horse has already bolted.

## Concluding remarks

In this paper, we have argued that the noetic account of SP, which suggests that progress in science tracks understanding rather than knowledge, is undermotivated. It is undermotivated because the examples which Dellsén uses to support the idea that understanding is separable from knowledge fail in this purpose. We have shown that the cases of Alice, Bernie and Einstein are, in fact, (somewhat disguised) Comanche-style cases. As a result, these scenarios fall found of Grimm’s criticism of Kvanvig’s original Comanche case.

In Sect. 3, we saw that Grimm (2006) highlights two conflicting interpretations of such cases. They can be seen as either instances where knowledge and understanding are both present, or as instances in which neither notion is present. The problem for Kvanvig (and, as a result, Dellsén) being that neither interpretation supports the thesis that such cases can distinguish understanding from knowledge. In Sects. 4 and 5, we showed how Grimm’s argument can be applied to the Alice’s and Bernie’s cases yielding the same result: neither supports Dellsén’s position.

In Sects. 6 and 7, we showed that Einstein’s case is also susceptible to Grimm’s argument against Kvanvig’s Comanche case, rendering this scenario similarly silent with respect to a separation of understanding and knowledge. However, we also argued that Einstein’s case allows us to show that Grimm’s first interpretation of such Comanche-style cases is the correct one.

Through a comparison with a recent study by Lourenço et al. (2020), we argued that neither understanding nor knowledge ought to be attributed to Einstein in 1905, because the accuracy of the explanatorily/predictively essential elements of his theory were not known, and SP (in both cases) appears to be intuitively tied to those episodes with bring about such knowledge. What is more, by tying SP to understanding, the noetic account appears to jump the gun with respect to these episodes, being incapable of making sense of the clear sense in which they are progressive.

## Footnotes

1 See e.g., Achinstein (1983), Salmon (1989), Kitcher (2002), Woodward (2003), Lipton (2003) and Bird (2007).

2 Here, Grimm cites: Elgin (1996, 2004), Zagzebski (2001); and Kvanvig (2003). We could now expand this list to include (among others): Elgin (2007), Pritchard (2008), Kvanvig (2009), Gardiner (2012); and Mizrahi (2012).

3 For examples not covered in detail here, see de Regt (2015, 2016) and Wilkenfeld (2017, 2019).

4 While antirealist notions of SP are available, most notably the *functionalist-internalist* accounts of Kuhn (1962, 1991) and Laudan (1977, 1981, 1984), interest in them has waned in recent years; Shan (2019) is a notable exception.

5 The connection between truth and progress is most famously highlighted in Putnam’s (1975) ‘no miracles’ argument, a more contemporary interpretation of which can be found in Lipton (2003). Although, not all realists agree that arguments of this form are convincing; see Bird (2021) for example.

6 Also see Mizrahi (2013, 2017); and Mizrahi (2017), Park (2017), and Sterpetti (2018).

7 Rowbottom (2008), for example, argues that justification is merely *instrumental* to progress; and Cevolani and Tambolo (2013) argue that the semantic conception can handle issues of justification by means of a distinction between “real” and “estimated” progress without needing to concede that SP is the accumulation of scientific knowledge.

8 In “Understanding without justification or belief” Dellsén notes that it is common to distinguish between different kinds of understanding ‘some of which are not relevant to epistemology and thus clearly would not replace knowledge as the primary focus of the field’ (2017: p. 2, fn.1). *Objectual understanding* is understanding that is grammatically followed by an object, ‘as in ‘she understands economic depression’ (Dellsén, 2017: p. 2, fn.1). It is this form of understanding which is of central interest to Dellsén and as a result, to *us*. However, Dellsén notes that ‘what I have to say will apply *mutatis mutanda* to understanding-why as well’ (2017: p. 2, fn.1). For a detailed account of the distinction between understanding-why and objectual understanding see e.g., Khalifa (2013).

9 According to de Regt, understanding is a matter of *intelligibility*; being able to use and manipulate a model ‘in order to make inferences about the system, to predict and control its behaviour’ (2015: p. 3791). Since this notion of understanding does not track truth, an account of SP based upon it would provide a natural alternative to traditional, antirealist, functionalist-internalist accounts of SP, although de Regt does not pursue this line of enquiry (see fn. 1 for more detail). More recently still, Wilkenfeld (2017, 2019) has advocated a similar position.

10 While some epistemologists have argued for the stronger position, that understanding requires *full-*factivity (e.g. Grimm, 2006; Pritchard, 2010; Hills, 2009), most follow Dellsén in taking the notion to be quasi-factive (e.g. Kvanvig, 2003, 2009; Mizrahi, 2012; Wilkenfeld, 2017).

11 In this sense, Dellsén admits that his noetic account of SP is remarkably similar to the semantic account (2018: p. 10). The principal difference being ‘whether proposing new explanations or making new predictions could itself constitute progress, even when there is no change in the theories with which one would explain and predict’ (2018: p. 10). While Rowbottom (2015) has argued that the semantic view cannot accommodate such progress, Dellsén (2016, 2018) believes that his own account can.

12 Severing the truth or justification connections are not the only options available in distancing understanding from standard accounts of knowledge. There is, of course, a third notion involved: belief. Dellsén (2017) himself puts forward the idea that a notion of understanding based upon *acceptance* rather than belief would also suffice to distinguish knowledge from understanding. However, since the distinction between belief and acceptance plays little role in Dellsén’s (2016) account of scientific progress, we shall not discuss it any further.

13 It is worth noting that while Dellsén (2016, 2017) cites Kvanvig (2003, 2009) in reference to arguments which distinguish knowledge from understanding, he does not make any reference to structural similarities between his own examples and Kvanvig’s Comanche case; nor indeed Grimm’s (2006) subsequent criticism of it.

14 Indeed, as Grimm highlights in a footnote, if understanding could be acquired upon the basis of such slipshod way, it becomes difficult to see why disciplines such as sociology, economics and history would need to develop ‘their methodologies and established canons for constructing and evaluating explanation’ (Grimm, 2006: p. 526, fn.14).

15 As Dellsén notes: ‘Bernie’s justification for believing [the con man’s] testimony about the car, forms no part of the explanation of the car’s breaking down’ (2017: p. 9).

16 This claim is not, strictly speaking accurate. As an anonymous reviewer at this journal points out, the original proof of Pythagorean theorem was given not by Pythagoras, but by Euclid in the first book of his *Elements*. Indeed, the statement of the theorem was known (without proof) to the Babylonians long before either Euclid or Pythagoras were born. We take it that one could happily substitute ‘Euclid’ for ‘Pythagoras’ without any loss of content to the argument above. However, to avoid any confusion, we have ignored this minor historical inaccuracy in the main body of the text.

17 Wilkenfeld’s (2017) paper concerns de Regt’s (2015) antirealist notion of understanding, which was briefly mentioned in section II. As far as we are aware, no one has yet developed such considerations with respect to understanding as a characterization of scientific progress.

18 This story was originally published in *Financial Times* (see: https://www.ft.com/content/5ff6469a-6dd8-11ea-89df-41bea055720b), although it was later picked up by *The Times, Daily Express, Evening Standard, Daily Mail* and *The Sun.* It is important to note that, at time of writing, this research has still yet to be published, or even peer-reviewed. In an interview with *Wired* (see: https://www.wired.co.uk/article/coronavirus-infections-oxford-study-immunity?intcid=inline_amp), Colbourn (2020) highlights that ‘[i]t is a little concerning that they’ve taken it straight to the media… [i]t has not been properly sense-checked against any data’. It is precisely this unusual situation which makes this case-study fit for our purposes.

19 There may well be room for debate upon this point although, as Dellsén himself highlights, ‘we can easily imagine a world in which Einstein’s explanation was put forward before the kinetic theory of heat became sufficiently justified to be known (e.g. shortly after James Clerk Maxwell first presented his kinetic theory in 1859)’ (2016: p. 76).
